# Cell-mediated immune responses to different formulations of a live-attenuated tetravalent dengue vaccine candidate in subjects living in dengue endemic and non-endemic regions

**DOI:** 10.1080/21645515.2019.1581536

**Published:** 2019-04-15

**Authors:** Philippe Moris, Kristen M. Bauer, Jeffrey R. Currier, Heather Friberg, Kenneth H. Eckels, Ines O. Esquilin, Robert V. Gibbons, Bruce L. Innis, Richard G. Jarman, Sriluck Simasathien, Peifang Sun, Stephen J. Thomas, Veerachai Watanaveeradej

**Affiliations:** a GSK, Rixensart, Belgium; b Landstuhl Regional Medical Center, Landstuhl, Germany; c Viral Diseases Branch, Walter Reed Army Institute of Research, Silver Spring, MD, USA; d Pilot Bioproduction Facility, Walter Reed Army Institute of Research, Silver Spring, MD, USA; e Department of Pediatrics, University of Puerto Rico School of Medicine, San Juan, Puerto Rico; f Battlefield Pain Management Task Area, U.S. Army Institute for Surgical Research, Fort Sam Houston, TX, USA; g GSK, King of Prussia, PA, USA; h Department of Pediatrics, Phramongkutklao Hospital, Bangkok, Thailand; i Henry Jackson Foundation for the Advancement of Military Medicine, Bethesda, MD, USA; j Department of Microbiology, Phramongkutklao College of Medicine, Bangkok, Thailand

**Keywords:** Live-attenuated tetravalent dengue candidate vaccine, Cellular-mediated immune responses, Dengue-primed and -unprimed populations

## Abstract

Three phase II randomized trials evaluated the safety/immunogenicity of two formulations of live-attenuated tetravalent dengue virus (TDEN) vaccine in dengue-endemic (Puerto Rico, Thailand) and non-endemic (US) regions (NCT00350337/NCT00370682/NCT00468858). We describe cell-mediated immune (CMI) responses; safety and humoral responses were reported previously. Participants received two doses of vaccine or control (placebo or the precursor live-attenuated TDEN vaccine) 6 months apart. Selected US participants received a booster 5–12 months post-dose 2. Evaluated subsets of the per-protocol cohorts included 75 primarily dengue virus (DENV)-unprimed US adults, 69 primarily flavivirus-primed Thai adults, and 100 DENV-primed or DENV-unprimed Puerto Rican adults/adolescents/children. T-cell responses were quantified using intracellular cytokine staining (ICS; DENV-infected cell-lysate or DENV-1/DENV-2 peptide-pool stimulation) or IFN-γ ELISPOT (DENV-2 peptide-pool stimulation). Memory B-cell responses were quantified using B-cell ELISPOT. Across populations and age strata, DENV serotype-specific CD4^+^ T-cell responses were slightly to moderately increased (medians ≤0.18% [ICS]), DENV-2–biased, and variable for both formulations. Responses in unprimed subjects were primarily detected post-dose 1. Response magnitudes in primed subjects were similar between doses. Multifunctional CD8^+^ T-cell responses were detected after peptide-pool stimulation. T-cell responses were mostly directed to DENV nonstructural proteins 3 and 5. Memory B-cell responses were tetravalent, of low-to-moderate magnitudes (medians ≤0.25%), and mainly observed post-dose 2 in unprimed subjects and post-dose 1 in primed subjects. A third dose did not boost CMI responses. In conclusion, both formulations of the live-attenuated TDEN vaccine candidate were poorly to moderately immunogenic with respect to B-cell and T-cell responses, irrespective of the priming status of the participants.

**Abbreviation** ATP: according-to-protocol; ICS: Intracellular Cytokine Staining; NS3: Nonstructural protein 3; ELISPOT: Enzyme-Linked ImmunoSpot; JEV: Japanese encephalitis virus; PBMC: peripheral blood mononuclear cells

## Introduction

Dengue is endemic in over 125 countries in tropical and sub-tropical areas. There are an estimated 390 million dengue infections annually worldwide, and in recent years 50–100 million infections manifested clinically each year.^1-3^ The causative agents, the dengue viruses (DENVs), are single-stranded, positive-sense RNA viruses of the *Flaviviridae* family. Infection with any of the four antigenically distinct, but closely related serotypes (DENV-1, −2, −3 and −4) can manifest as a subclinical infection, dengue fever, dengue hemorrhagic fever, or dengue shock syndrome.^4^

Correlates of risk or protective immunity have not been established for dengue.^5^ Neutralizing antibody (nAb) responses (naturally induced) have been associated with protection from infection with DENV-1, −2 and −4, with DENV-2 potentially requiring higher titers.^6^ The nAb responses have partial cross-reactivity between serotypes. Infection-induced homotypic immunity can confer long-term protection, potentially persisting for decades, while the induced heterotypic responses and their protective capacities are thought to wane after a few years.^7^ It has been proposed that during a secondary infection, pre-existing heterotypic non-neutralizing antibody profiles could be associated with increased dengue immunopathology.^8^ However no direct association between nAb response profiles (with respect to serotype specificity and neutralization titers) and protection or risk of disease was observed in efficacy trials evaluating a live-attenuated tetravalent dengue (TDEN) vaccine, at least in the available sample set.^9^

Cell-mediated immune (CMI) endpoints, and their associations with protection or pathogenicity, have also been explored in multiple clinical vaccine trials. Though such associations were generally weaker as compared to those for humoral responses, the cell-mediated arm of the immune system is thought to contribute to protection against dengue. DENV serotype-specific memory B cells are important for the secondary humoral response. In addition, high-frequency, pre-existing T-helper (Th) and cytotoxic T cells (together with antibodies) are believed to mediate protection by neutralizing the virus, secreting inflammatory cytokines (notably IFN-γ) and killing infected cells.^10-14^ Studies in a transgenic murine model deficient in IFN-α/β and IFN-γ receptors support a protective role for T cells against primary infection (reviewed in ref.^15^). Yet, while high-avidity, homologous, tetravalent and Th1-biased memory T-cell responses activated after secondary infection are considered beneficial, low-affinity, heterologous and TNF-α–biased memory T-cell responses might promote immunopathology.^16^ Moreover, clinical outcomes may depend on the epitope(s) targeted by the DENV-specific T-cell response.^17,18^

One live-attenuated recombinant vaccine (Dengvaxia, Sanofi Pasteur) has been registered in several countries with an indication for vaccination in those aged 9 years and older, but its efficacy varies across the infecting serotype(s) and the recipients’ pre-vaccination dengue serostatus.^7^ Furthermore, a safety signal (an increased risk of hospitalization for dengue disease) was observed in vaccine recipients 2–5 years of age.^19,20^ Therefore, a dengue vaccine that is efficacious for all ages and independent of serotype or prior exposures to DENV remains a critical medical need. Several other live-attenuated dengue vaccine candidates are in development.^21^

The precursor of the TDEN vaccine candidate, F17/Pre, consisting of monovalent vaccines combined into a tetravalent preparation at administration^22,23^ was evaluated in phase I/II pediatric studies in Thailand.^24,25^ Subsequent efforts to improve the vaccine’s quality led to two new formulations, F17 and F19. These formulations were prepared from re-derived, amplified F17/Pre master seeds and then lyophilized as a tetravalent vaccine.^26^ F17 and F19 differed only in their DENV-4 concentration. These formulations were evaluated in populations in a region where DENV is not endemic (US), as well as in DENV-endemic regions (Thailand and Puerto Rico).^26-28^ The study populations consisted of primarily DENV-unprimed US adults, primarily flavivirus-primed Thai adults, and a mixed-age Puerto Rican population that was balanced in terms of DENV-primed and -unprimed subjects. When administered in two doses 6 months apart, the vaccines were well tolerated and immunogenic in terms of nAb responses in each population, without consistent immunogenicity differences between the formulations across the three studies.

It is well established that the magnitude of DENV-specific immune response to a live-attenuated TDEN vaccine may vary by the subject’s baseline priming status with respect to DENV or other flaviviruses.^7,14,29^ While a majority of the primed subjects in the abovementioned trials mounted tetravalent nAb responses after a single dose, most of the unprimed subjects needed a second dose to attain this.^26-28^ Here, we report on the CMI responses observed in these trials. We characterized the DENV serotype-specific CD4^+^ and CD8^+^ T-cell and memory B-cell responses in subsets of the per-protocol cohorts, and investigated the role of DENV- and/or flavivirus-specific pre-existing immunity in the induced responses.

## Results

### Study populations

In the evaluated subsets of the ATP cohorts, 19%, 96%, and 61% of the US, Thai, and Puerto Rican subjects, respectively, were DENV- or flavivirus-positive in neutralization tests at baseline, reflecting the overall priming status of the full ATP cohorts (Table 1). In the Puerto Rican study 85% of the subjects in the Adult/Adolescent (≥12 years of age) subgroup were DENV-primed, while the distribution was more balanced in the Child (<12 years of age) subgroup in which 49% of the subjects were primed.

**Table 1.** Baseline immune status in the according-to-protocol cohorts and subsets.

Study		US			Thailand			Puerto Rico								
		Adults			Adults			Child (< 12 yoa)			Adult/Adolescent(≥ 12 yoa)				All	
		total	primed^#^		total	primed*		total	primed^#^		total	primed^#^		total	primed^#^	
Subjects	Treatment	N	n	%	N	n	%	N	n	%	N	n	%	N	n	%
ATP cohort	F17/Pre	20	4	20	-	-	-	-	-	-	-	-		-	-	
	F17	20	4	20	39	34	87	27	14	52	19	16	84	177	91	51
	F19	18	3	17	39	38	97	28	10	36	16	14	88	155	94	61
	Placebo	19	3	16	40	37	93	29	15	52	16	15	94	175	101	58
	*Total*	*77*	*14*	*18*	*118*	*109*	*92*	*84*	*39*	*46*	*51*	*45*	*88*	*507*	*286*	*56*
T-cell analysis (ICS^@^)	F17/Pre	20	4	20	-	-	-	-	-	-	-	-		-	-	
	F17	19	4	21	24	23	96	22	11	50	11	9	82	33	20	61
	F19	17	3	18	22	21	96	23	7	30	12	10	83	35	17	49
	Placebo	19	3	16	23	22	96	21	14	67	11	10	91	32	24	75
	*Total*	*75*	*14*	*19*	*69*	*66*	*96*	*66*	*32*	*49*	*34*	*29*	*85*	*100*	*61*	*61*
B-cell analysis	F17/Pre	18	3	17	-	-	-	-	-	-	-	-		-	-	-
	F17	19	4	21	12	-	-	-	-	-	18	15	83	-	-	-
	F19	15	2	13	8	-	-	-	-	-	15	14	93	-	-	-
	Placebo	17	3	18	-	-	-	-	-	-	15	14	93	-	-	-
	*Total*	*69*	*12*	*17*	*20*	-	-	-	-	-	*48*	*43*	*90*	-	-	-

ATP, according to protocol. yoa, years of age. ICS, intracellular cytokine staining. N, number of subjects with available results for the respective analysis at baseline (numbers may vary by the DENV serotype analysed). ^#^Seropositivity rate to DENV was defined as the percentage of subjects with neutralizing antibody (nAb) titers to at least one DENV serotype ≥ 1:10 dilution at pre-vaccination. *Seropositivity rate to DENV or Japanese encephalitis virus (JEV) was defined as the percentage of subjects with nAb titers to at least one DENV serotype or JEV ≥ 1:10 dilution at pre-vaccination. ^@^Numbers are indicated for the ICS assay using infected cell lysates, the ICS assay using peptide-pool stimulation and the IFN-γ ELISPOT assay described in the current report were performed for 15 US subjects (of whom 13 were vaccinated) and 13 US subjects (of whom 11 were vaccinated), respectively.

### CMI responses

#### T-cell responses measured by ICS assay

Different assays were performed (Table 2). DENV serotype-specific immune marker (CD40L/IL-2/TNF-α/IFN-γ)-expressing CD4^+^ and CD8^+^ T cells were first assessed using flow cytometry-based ICS assay upon *in vitro* stimulation with inactivated DENV-infected cell lysates. The gating strategy and representative scatterplots are presented in Figure S1.

**Table 2.** T- and B-cell assays.

		Study			
				Puerto Rico	
Assay	Stimulation/Antigen	US	Thailand	Child	Adult/Adolescent
ICS	Inactivated DENV-1,-2,-3 and −4-infected cell lysates*	+	+	+	+
	DENV-1 and −2 NS3 and E peptide pools	+			
IFN-γ ELISPOT	DENV-2 NS1, NS2, NS3, NS4, NS5, C, PrM, E peptide pools	+			
B-cell ELISPOT	DENV-1,-2,-3 and −4 recombinant truncated 80E	+	+		+

*This intracellular cytokine staining (ICS) assay was whole blood-based for the Child subgroup and based on peripheral blood mononuclear cells for the other subjects. DENV, dengue virus. PrM, premembrane. NS, nonstructural. E, envelope. C, capsid.

##### Children

For both vaccines, frequencies of DENV-specific CD4^+^ T-cells expressing at least one marker were low (medians ≤0.03%), and primarily DENV-2–biased after each dose, expressing IL-2 as well as IFN-γ and TNF-α (Figure 1(a)). Between the doses, the DENV-2–specific responses tended to be higher following the first dose of F17 or F19 in primed subjects (peaking at months 3 and 6, respectively), and higher following the second dose (M7) of F17 in unprimed subjects (Figure 1(b)). The latter trend was less clearly seen in the F19 group. No T-cell or B-cell responses were observed after administration of placebo (data not shown).

**Figure 1.**
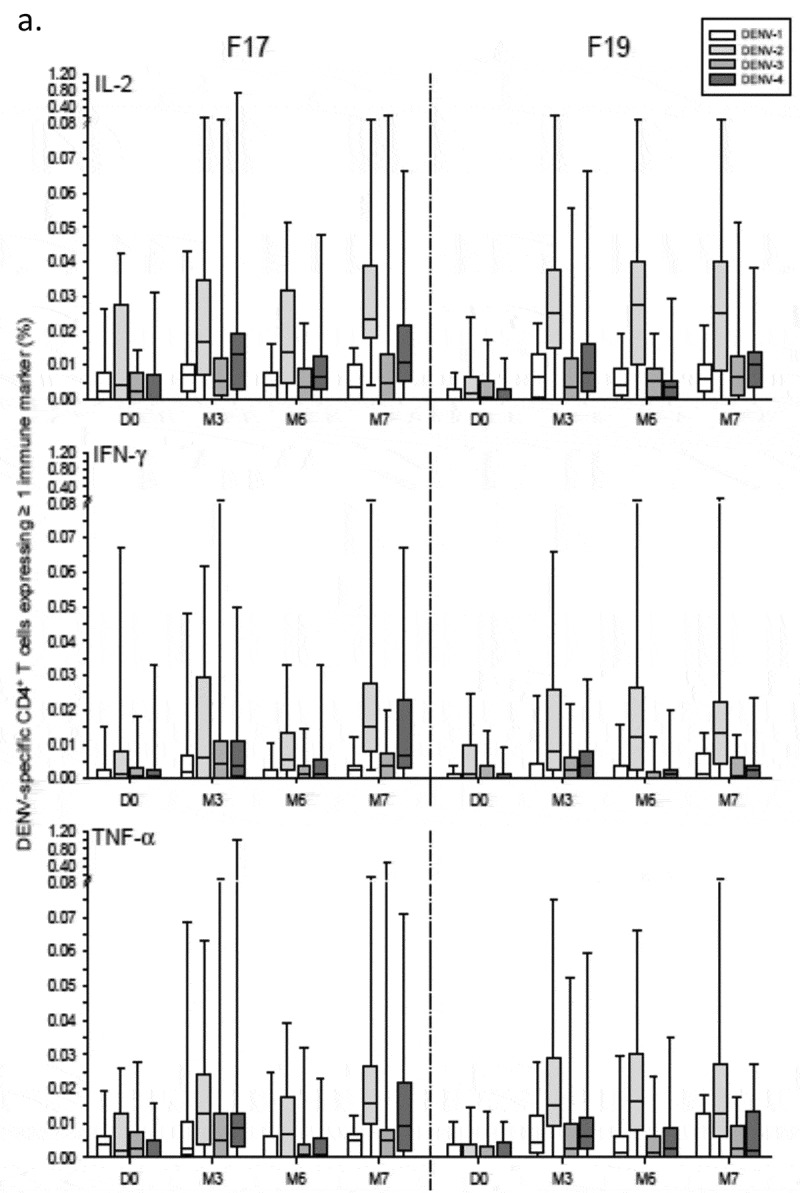
DENV-specific CD4^+^ T-cell responses in Puerto Rican children. DENV-specific CD4^+^ T-cell responses in Puerto Rican children aged 1–12 years after immunization with the F17 or F19 vaccines are presented for the overall sample subset (a), and stratified by their DENV priming status at baseline (b). Blood samples were obtained prior to each vaccination (Day [D] 0 and month [M] 6), 3 months after the first dose (M3) and 1 month after the second dose (M7). Data are represented in box-and-whiskers plots as percentages of DENV-specific CD4^+^ T cells expressing (after *in vitro* stimulation) at least IL-2, IFN-γ or TNF-α among all CD4^+^ T cells, with medians, first and third quartiles, and minimum/maximum values presented.

**Figure 1.**
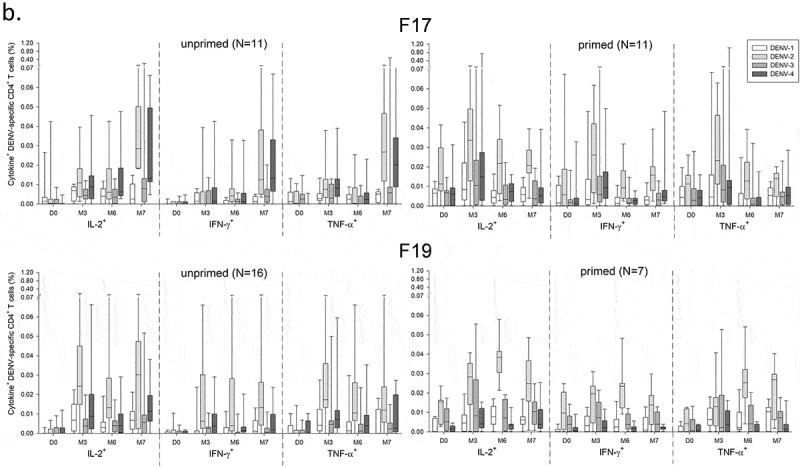
(Continued).

##### Adults and adolescents

Across the three studies, median frequencies of CD4^+^ T cells expressing at least two immune markers were increased (peak response ≤0.18%) when targeted to DENV-2, but very low (typically ≤0.04%) when targeted to the other serotypes (Figure 2(a)). No pre-vaccination responses were observed in the majority of US adults, consistent with their predominantly unprimed status. In these subjects, DENV-2–specific responses in the F17 and F19 groups were moderately increased after the first dose (month 1; medians ≤0.18%), with a relatively high inter-subject variability. These responses were decreased just before dose 2 (month 6), and marginally increased after dose 2 (month 7). No increased CD4^+^ T-cell responses were observed one month after the booster dose of F17 or F19 administered 5–12 months post-dose 2 (data not shown). However, it is noted that the number of subjects who received a third dose was low (N = 9 and 12 in the F17 and F19 groups, respectively). In addition, no consistent differences in responses were detected between the F17 or F19 vaccines on the one hand, and the precursor vaccine F17/Pre on the other hand (data not shown).

**Figure 2.**
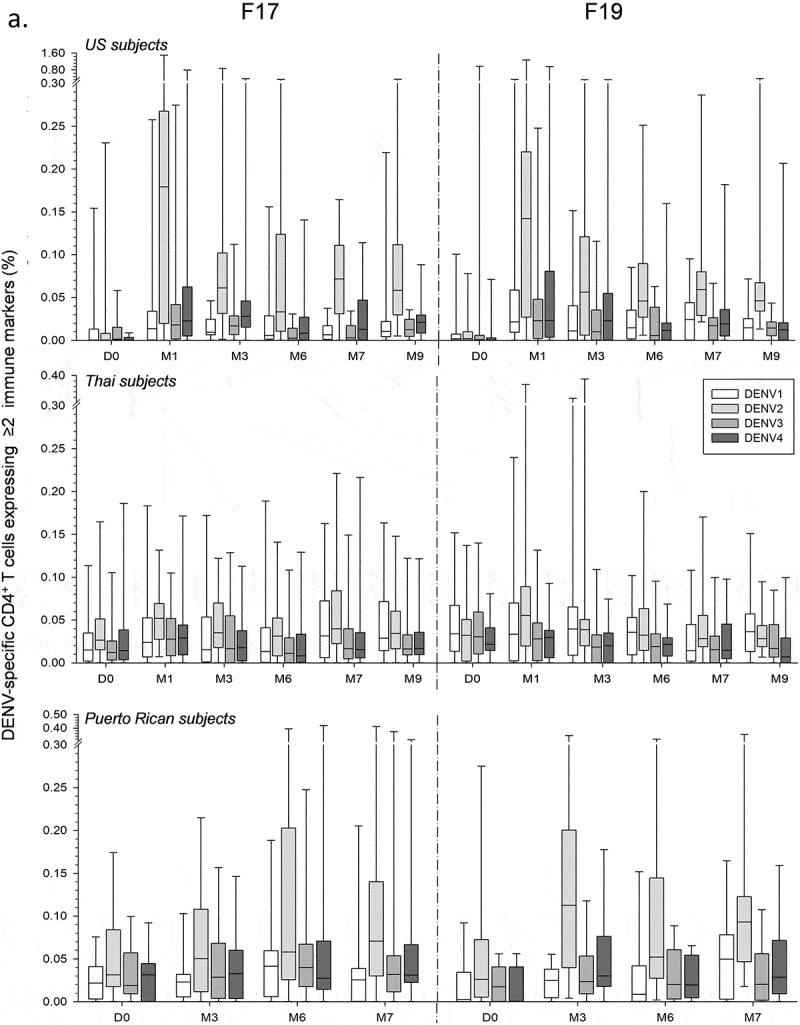
DENV-specific CD4^+^ T-cell responses in adults and/or adolescents in the US, Thailand and Puerto Rico. DENV-specific CD4^+^ T-cell responses after immunization with the F17 or F19 vaccines are presented for the overall sample subset of adults in the US and Thailand, and adolescents and adults in Puerto Rico (a), and for US subjects stratified by their DENV priming status at baseline, with numbers of evaluated subjects as indicated (b). Blood samples were obtained prior to each dose (Day [D] 0 and month [M] 6), 1 and 3 months after both the first dose (M1 and M3) and the second dose (M7 and M9). Data are presented in box-and-whiskers plots as percentages of DENV-specific CD4^+^ T cells expressing (after *in vitro* stimulation) at least two immune markers among IFN-γ, IL-2, TNF-α and CD40L of all CD4^+^ T cells, with medians, first and third quartiles, and minimum/maximum values presented.

**Figure 2.**
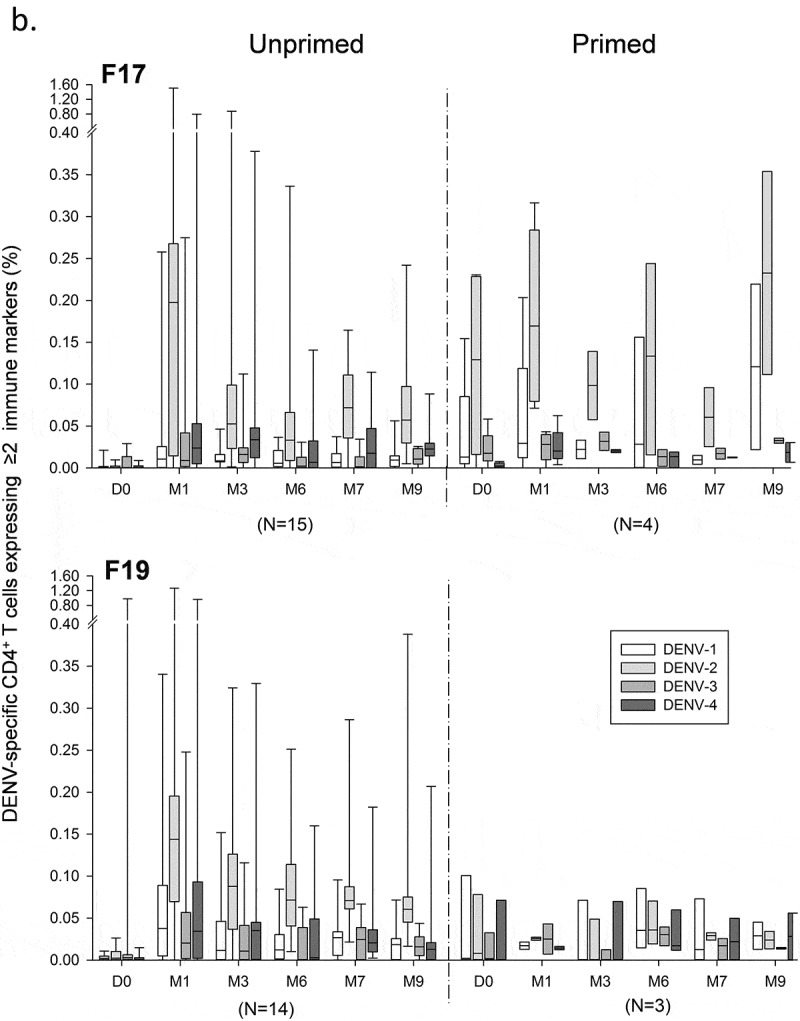
(Continued).

The majority of Thai adults had a baseline multitypic response, of which only the DENV-2–specific responses were marginally increased (medians ≤0.06%) after the first, but not the second dose of either vaccine. Low baseline responses were also seen in the Puerto Rican adults and adolescents, of which the DENV-2–specific responses were slightly increased after the first dose (medians ≤0.11%), while no or minor increases were observed after the second dose of F17 or F19, respectively. Further analysis of the DENV-2–specific responses in this population revealed that the cells responding to F19 expressed mostly IL-2 and IFN-γ, suggestive of a Th0/Th1-biased phenotype, whereas no consistent pattern was observed for cells responding to F17 (Figure S2).

No CD4^+^ T-cell responses were detected after administration of placebo in all three studies (data not shown).

As expected given the subset’s composition, stratification of the responses in US subjects by their priming status revealed that in both vaccine groups, the kinetics of the response in unprimed subjects followed that observed for the entire subset (Figure 2(b)). In contrast, the response kinetics in primed subjects appeared similar to that seen in the Thai subjects, with no or only minor increases after each dose. Yet, no definitive conclusions could be drawn due to the low sample sizes of primed subjects (N = 3 or 4 *vs* N = 14 or 15 for unprimed subjects).

**Figure 3.**
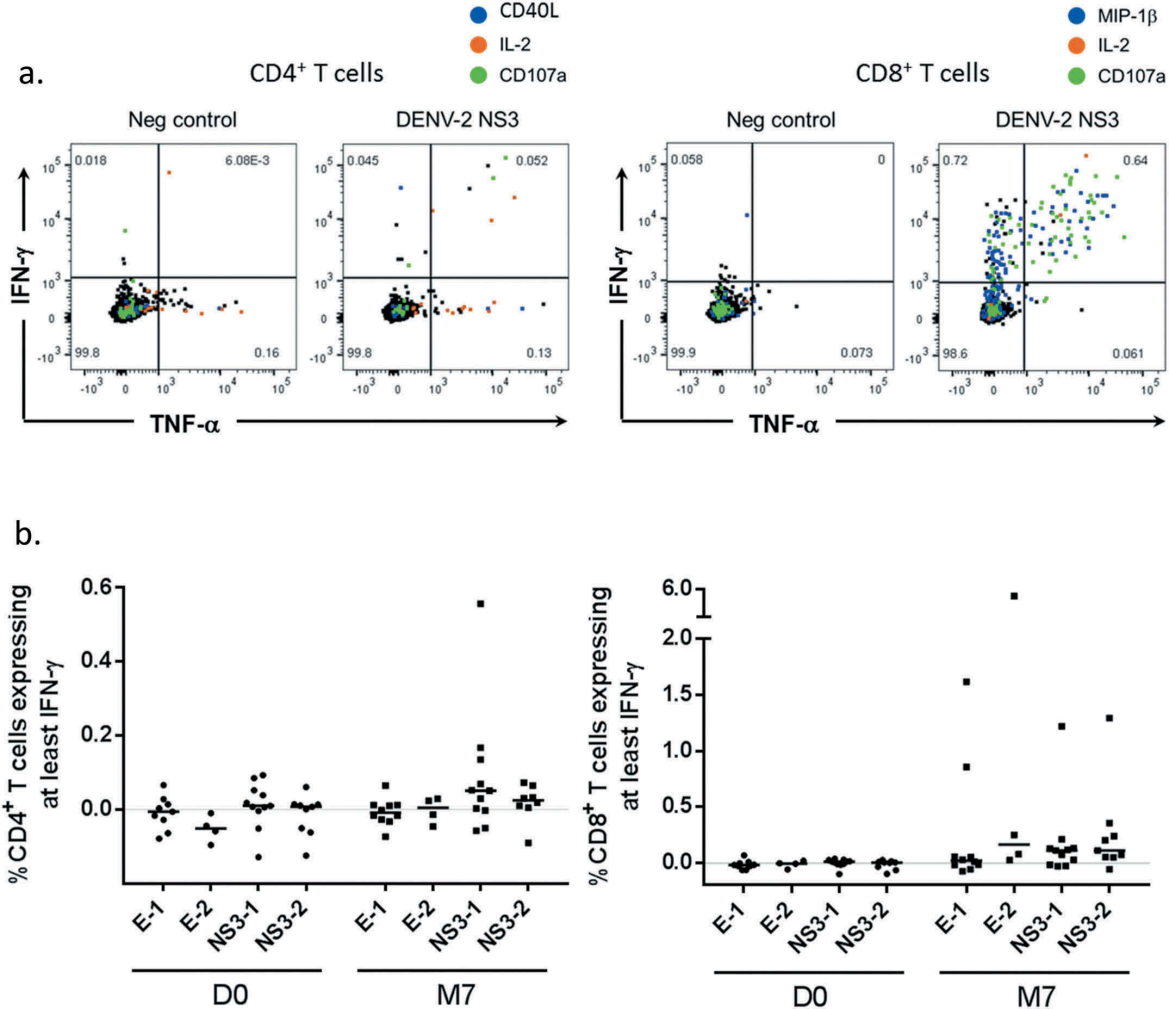
DENV-specific T-cell responses in US adults (ICS with peptide pool stimulation). (a) Representative flow plots of IFN-γ *vs* TNF-α production by CD4^+^ T cells or CD8^+^ T cells (left-hand and right-hand panels, respectively) after stimulation of peripheral blood mononuclear cells with negative control or the DENV-2 nonstructural (NS) 3 peptide pool are shown. Cells co-expressing other functions (CD107a, IL-2, CD40L or MIP-1β) are highlighted in color. Data are from one representative subject at 1 month after the second dose (M7). (b) Magnitudes of responses of CD4^+^ T cells and CD8^+^ T cells (left-hand and right-hand panels, respectively) producing at least IFN-γ after stimulation with DENV-1 envelope (E), DENV-2 E, DENV-1 NS3, or DENV-2 NS3 peptide pools are presented for 13 vaccinated subjects. PBMC were obtained pre-vaccination (D0) and 1 month after the second dose (M7). Data are background (negative control value)-subtracted. For the subjects with a D0 value available, a response was considered positive if it was ≥0.05%, and the background-subtracted value at M7 was at least 3 times higher than the subject-matched value at day 0.

No CD8^+^ T-cell responses were detected in these ICS assays (data not shown). This may be a consequence of the nature of the stimulating antigen used in this assay (see Table 2). Indeed, DENV-specific IFN-γ^+^ CD8^+^ T-cell responses, as well as IFN-γ^+^ CD4^+^ T-cell responses were detected 1 month post-dose 2 in the US study, using a modified ICS assay with DENV-1 and −2 peptide-pool stimulation (Figure 3(a)). Antigen-specific CD4^+^ and CD8^+^ T-cell responses were observed in 3 and 9 of the 13 vaccinated subjects tested, respectively (Figure 3(b)) but not in the two tested placebo recipients (data not shown). A considerable proportion of the responding CD4^+^ or CD8^+^ T cells exhibited multifunctional expression profiles (i.e., expressing ≥2 immune markers, among CD40L [for CD4^+^ T cells], IL-2, TNF-α, IFN-γ, the degranulation marker CD107a, and [for CD8^+^ T cells], MIP-1β, a marker of inflammatory phagocytes). The responding CD8^+^ T cells were targeted against any of the four tested antigens, while the CD4^+^ T cells were mostly targeted against DENV-1 NS3.

#### T-cell antigen specificity and cross-reactivity

**Figure 4.**
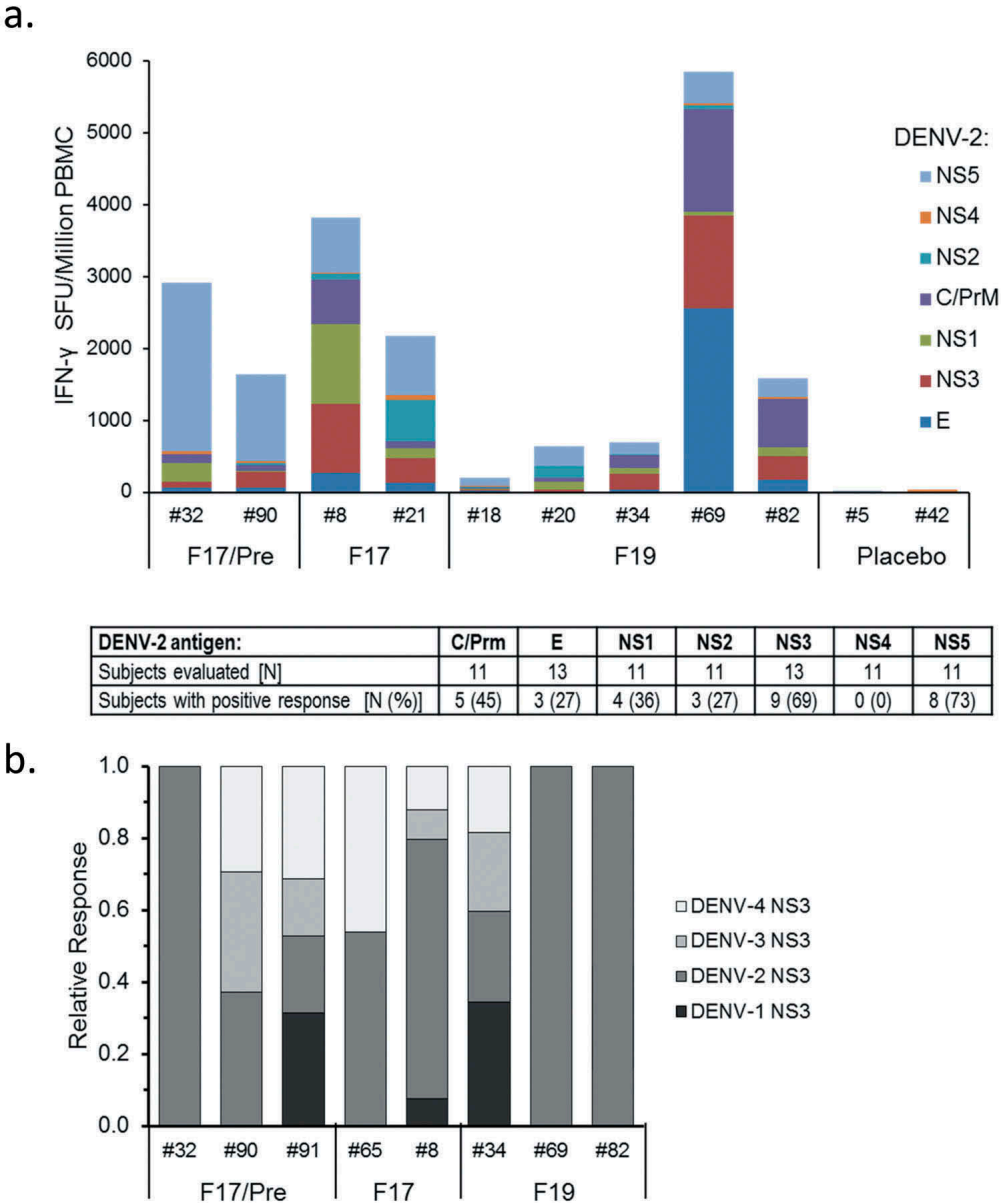
Specificity of T-cell responses in DENV-unprimed subjects. The magnitudes and specificity of background (medium)-subtracted T-cell responses to different DENV antigens measured at 1 month after the second dose (Month 7) by IFN-γ ELISPOT are presented. Samples were collected from a subset of US subjects (with subject numbers indicated) who were DENV-unprimed at baseline. Each bar represents the response of one subject. A response was considered positive if it was ≥55 spot-forming units (SFU) per 10^6^ PBMC, and exceeded response to the negative control by four-fold. (a) Magnitudes of T-cell responses to individual DENV-2 antigens measured after stimulation with peptide pools covering DENV-2 nonstructural (NS) 1, NS2, NS3, NS4, NS5, capsid (C), premembrane (PrM) and envelope (E) proteins are presented. (b) Relative proportions of the T-cell response to DENV-1, DENV-2, DENV-3 and DENV-4 NS3 peptide pools were determined for the subjects with DENV-2 NS3 specific response in the analysis shown in (a).

Next, the specificity of the induced T-cell response was investigated at the antigen level. For a subset of the unprimed US subjects, T-cell specificity for eight individual DENV-2 antigens was assessed in IFN-γ ELISPOT (see Table 2). No baseline responses were detected, consistent with the ICS results (data not shown). At 1 month post-dose 2, the majority of vaccine recipients mounted a response that was predominantly directed to NS3 and NS5 (detected in 82% and 73% of subjects, respectively), but the specificities were highly variable between subjects (Figure 4(a)). When the serotype cross-reactivity of the T cells was determined for eight subjects with a DENV-2 NS3-specific response, monovalent, bivalent, trivalent and tetravalent NS3-specific responses were observed in 3, 1, 1 and 3 subjects, respectively (Figure 4(b)), though the low sample size precluded any further conclusions.

#### Memory B-cell responses

Memory B-cell responses were measured using the B-cell ELISPOT assay (Figure S3). Across the three studies, frequencies of DENV-specific memory B-cell responses in adults or adolescents were low to moderately high (medians ≤0.25%), balanced, and relatively variable between the subjects (Figure 5(a)), while no responses were detected after administration of placebo (data not shown). Indeed, both doses of either vaccine induced a response in a minority of the US subjects, which particularly in the F19 group appeared to be higher after dose 2. Aligned with the CD4^+^ T-cell responses, we observed in this study no memory B-cell responses after the booster vaccinations with F17 and F19, nor any differences between these formulations and the F17/Pre control. Among the Thai subjects, by contrast, the majority of subjects in both groups elicited a response after the first dose, with lower inter-subject variability and higher median responses in the F19 group, while only a minority of the subjects in either group demonstrated a boost after the second dose. By comparison, most of the Puerto Rican subjects mounted only responses to the F17 vaccine, which were of comparable magnitudes between the doses, though the variability was also relatively high. Similar results were obtained when these frequencies were corrected for the baseline response by subject (Figure 5(b)). Evaluation by priming status in the US study revealed a tendency for a higher increase in baseline responses in the primed subjects (Figure 5(c)), though sample sizes were low and unbalanced between the primed/unprimed subgroups.

**Figure 5.**
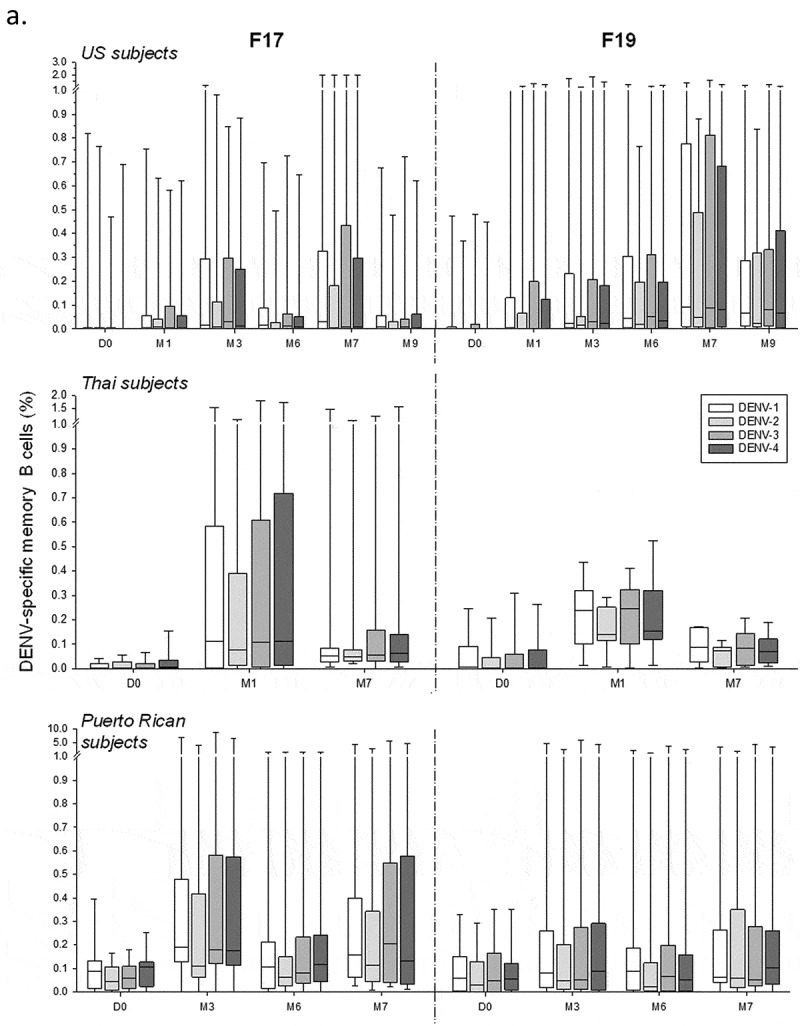
DENV-specific B-cell responses to tetravalent live-attenuated DENV vaccines in different study populations. (a) Frequencies of DENV-specific B-cells for the overall sample subsets of study populations in the US and Thailand (adults) and Puerto Rico (adults and adolescents) after immunization with F17 or F19 vaccine are presented. Blood samples were obtained prior to each dose (Day [D] 0 and month [M] 6), at 1 and 3 months after both the first dose (M1 and M3) and the second dose (M7 and M9). Box-and-whiskers plots represent the percentages of responding B cells with medians, first and third quartiles, and the minimum/maximum values measured. (b) B-cell responses in the Puerto Rican subjects after subtraction of the pre-vaccination responses by subject are presented. (c) B-cell responses in the US subjects stratified by their DENV priming status at baseline are presented, with numbers of evaluated subjects indicated (numbers may vary by time-point and serotype).

**Figure 5.**
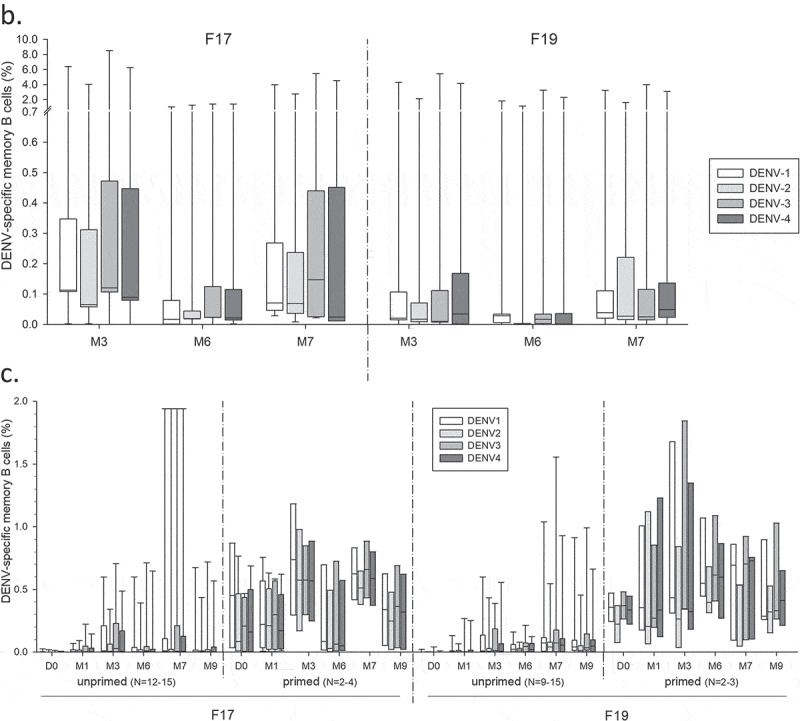
(Continued).

**Figure 6.**
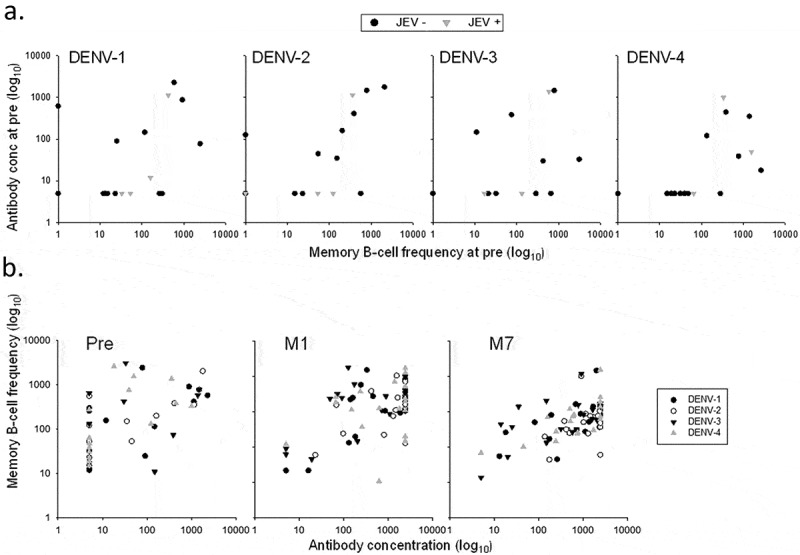
Correlations between memory B-cell responses and neutralizing antibody responses in Thai adults. Memory B-cell responses and DENV-specific neutralizing antibody responses observed in Thai adults are presented at pre-vaccination (pre), by the subjects’ baseline Japanese encephalitis virus (JEV) priming status (a), and at pre-vaccination, 1 month after the first (P1(M1)) and the second (PII(M7)) dose, by DENV serotype (b).

Among the Thai subjects, there was no apparent impact of the JEV priming status on the B-cell responses to either vaccine at baseline (Figure 6(a)), though again sample sizes were low and unbalanced. The B-cell response did however seem to correlate positively with the previously reported^27^ DENV-specific nAb responses at months 1 and 7 (Figure 6(b)).

## Discussion

We previously reported on the humoral immunogenicity of the F17/Pre, F17 and F19 formulations in different populations.^26-28^ As for other live-attenuated DENV vaccines,^14,29,30^ we observed that the absolute magnitudes of nAb responses in primed individuals were generally greater than in unprimed subjects, although the vaccine-induced increases from pre-vaccination responses were lower in primed *vs* in unprimed subjects. In the US subjects, nAb responses to the first dose were biased to one serotype (most often DENV-2) and boosted and broadened to the other serotypes after the second dose,^26^ whereas in the Thai subjects, the highest increase was observed after the first dose.^27^ These differences may stem from variations in B-cell and T-cell memory from prior infection(s), resulting in different effector responses to vaccination. Here, we sought to investigate the impact of baseline priming on the T-cell and memory B-cell vaccine-induced responses underlying the nAb responses.

This investigation makes the following points: (1) Administration of the F17 or F19 vaccines induced variable and primarily DENV-2–biased CD4^+^ T-cell responses and low to moderately increased tetravalent memory B-cell responses in each population, without consistent differences between the formulations; (2) Aligned with the nAb response,^26^ the third dose offered to the US adults did not boost the CMI response; (3) Upon evaluation in some individuals, the vaccines induced CD8^+^ T-cell responses with a multifunctional cytokine-expression profile; (4) While the trends in the kinetics of memory B-cell responses tended to mirror those seen in the nAb responses, no such parallel was apparent for the CD4^+^ T-cell responses.

Although most live-attenuated DENV vaccines are known to elicit mainly multitypic T-cell responses (reviewed in ref.^17^), in the present populations the induced CD4^+^ T-cell responses failed to develop into balanced multitypic responses and were mainly targeted against DENV-2. It is unknown whether this serotype hierarchy is associated with higher replication levels of the DENV-2 vaccine strain and/or lower replication levels of the other three vaccine viruses. Alternatively or in addition, stimulation of PBMC with inactivated DENV-infected cell lysates instead of peptide-pools may have rendered the ICS assay less reproducible, as previously suggested,^31^ and this could have prevented a consistent detection of multitypic responses.

In contrast to the nAb responses, the induced CD4^+^ T-cell responses in the unprimed (US) adults tended to be higher than in the primed adults/adolescents, and were increased mainly after the first dose. Possibly the absence of a pre-existing multitypic antibody response in these subjects allowed a more extensive replication of vaccine virus post-dose 1, inducing higher T-cell responses, while post-dose 2, replication was diminished by the immunity induced by the first dose. Similarly, in the Thai population, it is likely that the presence of pre-existing antibody responses limited viral replication, preventing a robust CMI response to vaccination. This was to some extent also the case in the Puerto Rican population, which was primed to a lower degree as compared with the Thai population. The lack of response to the third dose suggests that the long-term persisting humoral immunity sufficed to prevent vaccine virus replication after vaccination. Consistently, no vaccine-induced T-cell responses were detected in the majority of the Thai children who received a booster dose of F17/Pre or F17.^32^ When we compared the kinetics of CD4^+^ T-cell responses between the Child and Adult/Adolescent groups in the present study by priming status, the observed patterns tended to match when comparing primed subjects, but were variable when comparing unprimed subjects. Again, conclusions may be hampered by the difficulties inherent to the antigenic preparation used in our main assay.

We presume that the absence of detectable CD8^+^ T cells after stimulation of PBMC using inactivated DENV-infected cell lysates was related to the assay rather than to the vaccines’ specific immunogenicity, since the protein format of the antigens may have been suboptimal for the detection of CD8^+^ T cells.^10,33-35^ Indeed, we did detect CD8^+^ T-cell responses in the ICS assay with peptide-pool stimulation, similar to observations for other live-attenuated dengue or yellow fever vaccines using comparable methods.^36-39^ A possible explanation is that the inactivated viral antigen in the cell lysate is preferentially presented through the MHC-II pathway, while peptide pools of varying lengths may be presented by both MHC-I and MHC-II pathways, resulting in a different CD4^+^/CD8^+^ T-cell balance between the two stimulation methods.^33^

CD8^+^ T-cell responses to infection or live-attenuated DENV vaccines are often biased toward the highly conserved NS proteins, particularly NS3 and NS5, while infection-induced CD4^+^ T-cell and B-cell responses mainly target the structural proteins E, PrM and C, as well as NS1.^36,37,39-41^ In the present study, the DENV-2–specific T-cell responses in the US adults were mainly directed to NS3 and NS5 (it is noted that the assay used for this analysis did not allow identification of the T-cell phenotypes). Furthermore, a proportion of the responding CD4^+^ and CD8^+^ T-cells tended to be Th0/Th1-biased and multifunctional, as seen for other live-attenuated dengue vaccines in primed and flavivirus-unprimed subjects.^17^

Aligned with the nAb responses,^28^ memory B-cell responses in the Thai adults were generally higher than in the US adults, and increases were mostly detected post-dose 1 rather than post-dose 2 as seen in the US adults, likely associated with the difference in priming status of these populations. The putative correlation between the memory B-cell and nAb responses to vaccination in the Thai subjects may be explained by differentiation of the memory B cells into antibody-secreting plasma cells upon antigenic (re-)stimulation, even though for other viral pathogens it has been suggested that these cell populations may not be governed by the same regulating mechanisms.^42^

Since the vaccine-induced CMI responses were exploratory endpoints in these Phase II trials, the analyses were performed on randomly selected sample subsets of the per-protocol cohorts. The following limitations should therefore be noted in interpreting the current results. First, analysis of the difference in immunogenicity profiles between primed and unprimed participants was limited by the unbalanced distribution between these groups and relatively small sample sizes. Furthermore, due to these sample size limitations we were unable to stratify the primed subjects by their monovalent- or polyvalent-positive status at baseline, which may also have contributed to the substantial variability in CMI responses detected within each study population. For nAb responses, it has been observed that subjects with a baseline multitypic profile responded differently to live-attenuated TDEN vaccine administration as compared to seronegative or monotypic subjects,^43^ and this may also apply to the vaccine-induced CMI responses. Last, as noted earlier, adequate assessment of the CD8^+^ T cell responses was hampered by the use of inactivated DENV-infected cell lysates in the main ICS assay, and by the limited sample size in the ICS using DENV peptide pool-based stimulation.

The F17 formulation was previously shown to have a marginally stronger immunogenicity profile than F19 with respect to humoral responses,^28^ but this was not consistently seen for the CMI responses. Yet, in pediatric follow-up trials in Thailand, neither the first two doses of F17/Pre and F17, nor the booster vaccination with these vaccines administered 1 or 3.5 years later, induced durable multivalent nAb, B-cell or T-cell responses.^32^ This is consistent with the waning of CMI responses over the 5–12 months post booster vaccination as observed here with the F17 or F19 vaccines. While persistent and balanced nAb and CMI responses that are stable after several years are presumably key features of a successful dengue vaccine, interpretation of vaccine-induced immunity data is hampered by the limited understanding of the specific immune profile required for protection.^5^ This understanding is further complicated by the potential presence of asymptomatic dengue infections, as occasionally observed during vaccine trials,^25,27^ and by other issues which emerged from the efficacy trial data of the licensed dengue vaccine.^5,7,9^ Therefore, in the absence of identified immune correlates of risk or protection, these obstacles in dengue vaccine development can currently only be overcome by performing clinical endpoint studies, possibly supported by studies using dengue human infection models.^44^

## Conclusion

The live-attenuated TDEN vaccine candidates were poorly to moderately immunogenic with respect to B-cell and T-cell responses in primed and unprimed subjects. Two vaccine doses elicited mostly variable CMI responses, which could not be boosted by a third dose. While the B-cell responses were mostly tetravalent, T-cell responses were imbalanced between the serotypes. Previously the vaccine was shown to be unable to induce durable multivalent DENV-specific nAb responses.

A tetravalent purified inactivated DENV vaccine formulated with aluminum hydroxide or an Adjuvant System (AS01_E_ or AS03_B_) is currently in Phase 1 clinical trials (ref.^45^ and NCT01702857). Several scientific and technological challenges still have to be met to ensure the required development of future vaccines against this important medical need.

**Figure 7.**
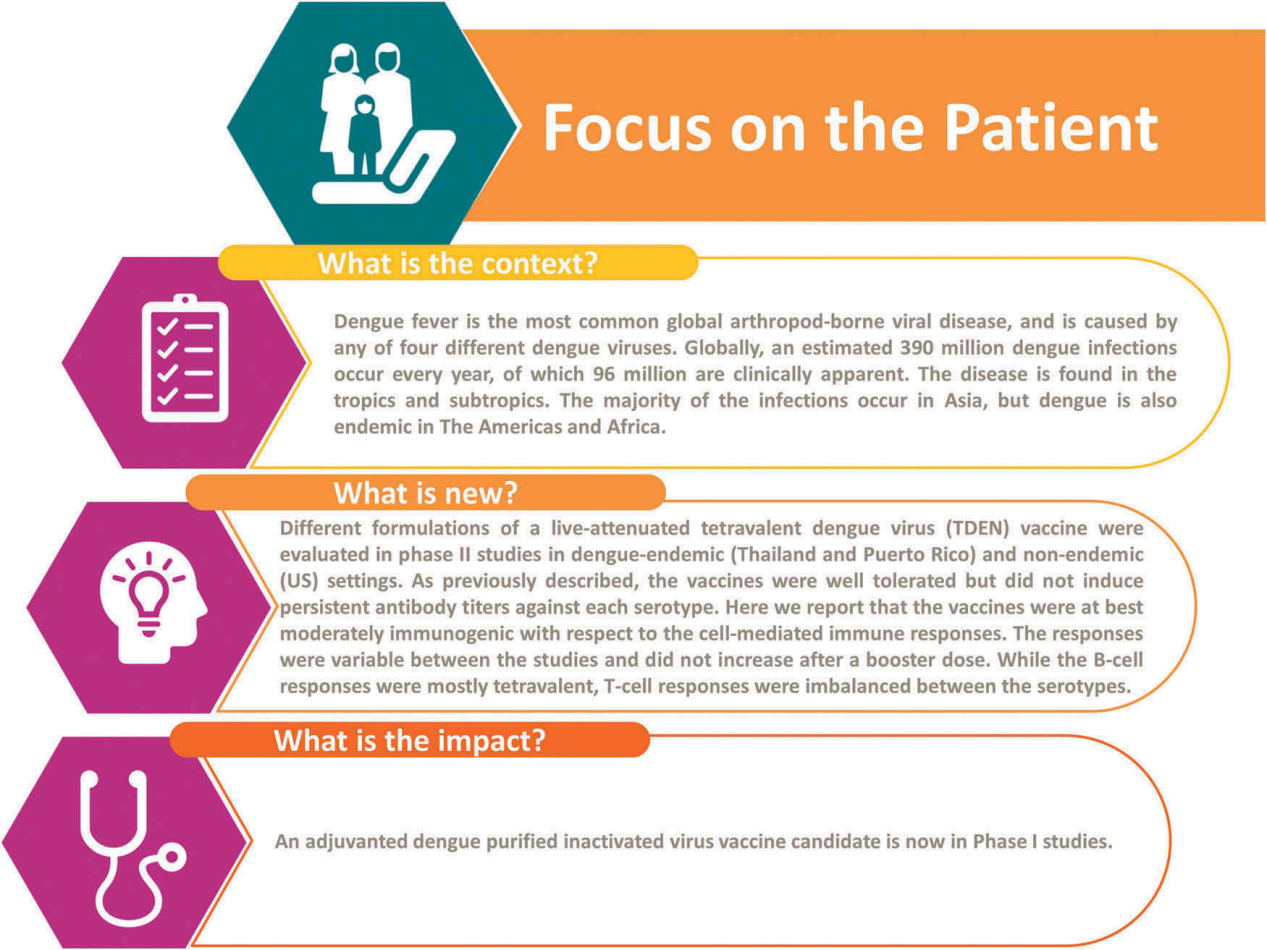
Focus on the patient section.

For the benefit of healthcare professionals, a graphical summary contextualizing the results and relevance of this clinical research is presented in Figure 7.

## Materials & methods

### Study designs

The placebo-controlled, randomized, phase II trials conducted in the US, Thailand and Puerto Rico (NCT00350337, NCT00370682 and NCT00468858, respectively; refs.^26-28^) evaluated the safety and immunogenicity of the F17 and F19 vaccines, with the precursor vaccine F17/Pre used as control in the US study. The studies were conducted in accordance with Good Clinical Practice guidelines, the 1996 Helsinki Declaration and respective state regulations.

### Objectives

The objective of the present manuscript was to describe DENV serotype-specific CD4^+^/CD8^+^ T-cell and memory B-cell responses to the F17, F17/Pre and F19 vaccines following the two-dose primary vaccination, and in the US adults also following the booster vaccination, in subsets of each study cohort.

### Participants

Healthy US, Thai, and Puerto Rican males and females were between 18 and 45, 20 and 25, and 1 and 50 years of age at enrollment, respectively. As described previously,^26-28^ US and Puerto Rican subjects were categorized at baseline as either DENV-primed (seropositive to at least one DENV serotype) or unprimed, and Thai subjects as either flavivirus-primed (seropositive to at least one DENV serotype or to JEV) or unprimed. The present evaluations were performed on sample subsets that were randomly selected from the ATPcohorts of each study (Table 1). For these evaluations, the subset of Puerto Rican subjects was stratified by age into the Child or Adult/Adolescent subgroups (ranges between 1 and 12 and ≥12 years of age, respectively).

### Vaccinations

F17/Pre contained four live-attenuated DENV strains (one each serotype).^25,46^ The two newer formulations F17 and F19 were prepared from re-derived F17/Pre vaccine strains following a slightly adapted process. The process modifications included: (1) application of three additional passages in fetal rhesus lung cells, (2) use of a carbohydrate stabilizer instead of human serum albumin for formulation of monovalent bulks, and (3) lyophilization of the final vaccine as a tetravalent product.^26^ F19 was identical to F17 except that its DENV-4 concentration was reduced by four-fold (US and Thai studies) or 50-fold (Puerto Rican study) relative to that in F17.^26-28^ Subjects received two doses of vaccine or cell-culture medium placebo 6 months apart. Nine and 12 of the US subjects of the F17 and F19 groups respectively received a third dose of the vaccine used for their primary immunization between 5 and 12 months after the second dose.

### Immunogenicity evaluations

Blood samples for CMI evaluations were collected before each dose (day 0, month 6), 3 months after the first dose (month 3), 1 month after the second dose (month 7), and 1 or 6 months after the third dose (for US subjects only). In the US and Thai studies, samples were also collected 1 month after the first dose (month 1) and 3 months after the second dose (month 9). Testing was performed at GSK, Rixensart, Belgium unless specified otherwise. Table 2 presents an overview of the CMI assays performed in each study.

#### T-cell responses

##### Intracellular cytokine staining

DENV serotype-specific CD4^+^/CD8^+^ T-cell responses in adults and adolescents were assessed using ICS and flow cytometry upon short-term *in vitro* stimulation of PBMC as described previously.^32,47^ For the Child subgroup in the Puerto Rican study, the ICS assay was performed using undiluted whole blood, based on a previously described assay.^48-50^ Briefly, PBMC or whole blood was stimulated for 2 h by a heat-inactivated (30 min, 56 °C) lysate of Vero cells infected with the respective DENV serotype (using the DENV-1–4 parent vaccine strains WP-74, S16803, CH53489, and 341750, respectively), or cultured with negative (medium) or positive (staphylococcal enterotoxin B) controls. Brefeldin A was then added for a subsequent 18-h incubation at 37°C. For whole blood samples, PBMC were isolated and fixed following the lysis of red blood cells and then frozen and stored at −80°C. The cells were subsequently stained and analyzed by flow cytometry, following a method that was similar to the one used for the PBMC samples.^32,47^ For both the PBMC-based and whole blood-based assays, results were expressed as percentages of antigen-specific CD4^+^/CD8^+^ T cells expressing combinations of the immune markers CD40L, IFN-γ, IL-2 and TNF-α. Background (the signals obtained in cells stimulated with medium only) was subtracted from all values. To evaluate DENV-specific CD4^+^/CD8^+^ T cells in adults and adolescents, ICS results were presented as background-subtracted percentages of CD4^+^ or CD8^+^ T-cells expressing at least two of the four immune markers. This parameter (instead of expression of a single marker) was selected to increase the specificity signal of the assay.

For a subset of the US subjects (N = 15; Table 1) an additional ICS assay was performed at the Walter Reed Army Institute of Research (WRAIR) laboratories (Silver Spring, MD, US). In this assay, stimulation was done with peptide pools covering the NS 3 and structural envelope (E) proteins of DENV-1 and DENV-2 serotypes. Briefly, cryopreserved PBMC were thawed and placed in RPMI 1640 medium supplemented with 10% FBS, L-glutamine, penicillin, and streptomycin (RPMI-10). Peptides (13 to 20-mer overlapping by 10–12 amino acids; BEI Resources) were resuspended in 100% dimethyl-sulfoxide (DMSO) at 200 µg/mL/peptide. PBMC were plated in 96-well plates at 1 × 10^6^ cells/well in a total volume of 200 µL RPMI-10, and stimulated with either 1 µg/mL/peptide of the relevant peptide pool in 0.5% DMSO (1:200 dilution), or medium (RPMI-10 containing 0.5% DMSO; negative control), in the presence of anti-CD28, anti-CD49d, and fluorescein isothiocyanate-conjugated anti-CD107a antibodies. Cells were incubated at 37°C for 1 h prior to addition of Brefeldin A and monensin, and were left overnight for further incubation at 37°C. Cells were then washed and stained with a viability marker followed by surface antibodies against CD3, CD4, CD8, CD14, and CD19. After fixation in 4% formaldehyde, cells were permeabilized and stained with antibodies against IFN-γ, TNF-α, macrophage inflammatory protein 1β (MIP-1β), IL-2, and CD40L, and analyzed. Results were expressed as background-subtracted percentages of antigen-specific CD4^+^/CD8^+^ T cells expressing combinations of the immune markers CD40L, IFN-γ, IL-2 and TNF-α, CD107a and MIP-1β. A response was considered positive if it was ≥0.05%, and the background-subtracted value at month 7 was at least three times higher than the subject-matched value at day 0.

##### IFN-γ ELISPOT

For a second subset of the US subjects (N = 13; Table 1) DENV-2–specific IFN-γ-secreting T cells were enumerated using IFN-γ ELISPOT as previously described.^51^ Testing was performed at WRAIR laboratories. Briefly, PBMC were stimulated *in vitro* overnight with DENV-2 peptide pools covering NS1, NS2, NS3, NS4, NS5, capsid (C), premembrane (PrM) and E. Human cytomegalovirus/Epstein-Barr virus/influenza virus (CEF) peptide pools were used as a positive control. A response was considered positive if it was ≥55 spot-forming units (SFU)/10^6^ PBMC and exceeded the response to the negative control (medium supplemented with 0.5% DMSO) by four-fold. Results were expressed as background-subtracted SFU/10^6^ cells. Serotype cross-reactive responses were evaluated for subjects with a DENV-2 NS3-specific response in the preceding analysis (N = 8). The NS3-specific response magnitudes were determined for each serotype using DENV-1–4 NS3 peptide pools.

##### Memory B-cell responses

DENV serotype-specific memory B-cell responses were quantified by B-cell ELISPOT as described previously^32^ (see Figure S3), and reported as frequencies of DENV antigen (recombinant truncated 80E protein)-specific memory B cells within the total memory B-cell population. No B-cell responses were assessed for the Child subgroup in the Puerto Rican study due to limited blood sample volumes.

### Statistical analyses

ICS data were analyzed using FlowJo software (FlowJo LLC, Ashland, OR, US) and GraphPad Prism (GraphPad Software, Inc., San Diego, CA). Frequencies of DENV-specific memory B cells and DENV-specific CD4^+^ and CD8^+^ T cells were calculated, and descriptive statistical analyses (including the calculation of medians, interquartile ranges, minima and maxima) of these responses were performed using SAS v9.1 (SAS Institute Inc., Cary, NC, US).
