# Supplementary material for: Cell-mediated immune responses to different formulations of a live-attenuated tetravalent dengue vaccine candidate in subjects living in dengue endemic and non-endemic regions
Source: Hum Vaccin Immunother. 2019 Apr 15;15(9):2090–105. doi: 10.1080/21645515.2019.1581536 (PMC6773406; doi:10.1080/21645515.2019.1581536)
Supplement: Supplemental Material [file khvi-15-09-1581536-s001.docx]

**Cell-mediated immune responses to different formulations of a live-attenuated tetravalent dengue candidate vaccine in subjects living in dengue endemic and non-endemic regions**

Philippe Moris, Kristen M. Bauer, Jeffrey R. Currier, Heather Friberg, Kenneth H. Eckels, Ines O. Esquilin, Robert V. Gibbons, Bruce L. Innis, Richard G. Jarman, Sriluck Simasathien, Peifang Sun, Stephen J. Thomas, Veerachai Watanaveeradej

**SUPPLEMENTARY MATERIAL**

**Fig. S1. Gating strategy for cell samples analyzed by cytometry**.


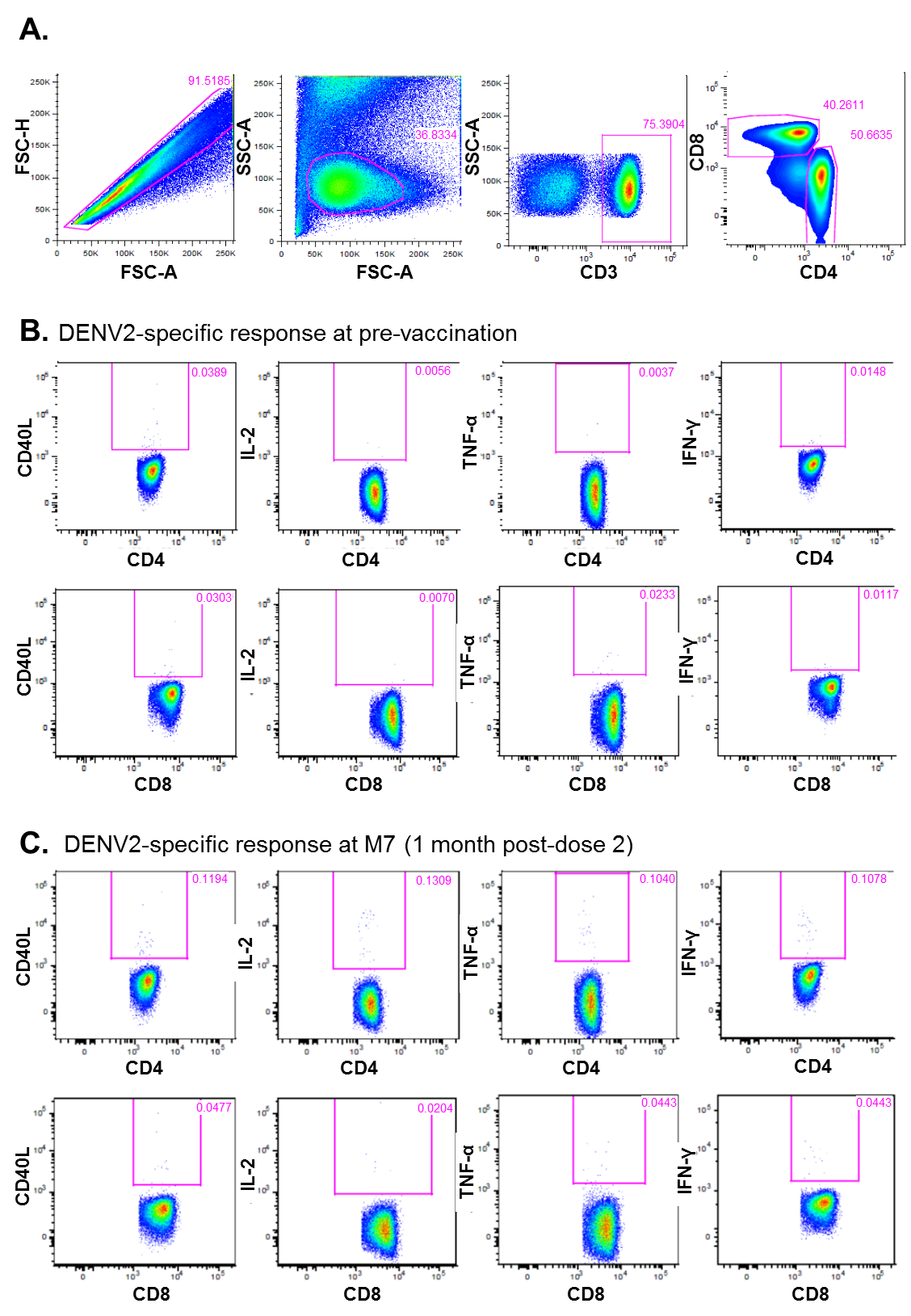


DENV serotype-specific immune marker (CD40L/IL-2/TNF-α/IFN-γ) expressing CD4^+^ and CD8^+^ T cells were assessed using ICS and flow cytometry upon *in vitro* stimulation with DENV-infected cell lysates. Data from a participant of the F19 vaccine group of the Puerto Rican study are shown. Results are representative of the range of responses seen for all participants. Numbers in pink font in the quadrant gates of the plots present each distinct population based on their marker expression. **(A)** The first gating allowed exclusion of doublets and aggregated cells (FSC-A, FSC-H), and was followed by gating on lymphocytes selected by size and granulometry (SSC-FSC), and then by gating on CD3^+^ events to subsequently identify CD4^+^ and CD8^+^ events. (**B and C)** Dotplots present the expression of CD40L, IL‑2, TNF-α and IFN‑γ by DENV-2-specific CD4^+^ (top row) and CD8^+^ (bottom row) T cells after *in vitro* stimulation with a preparation of DENV-2-infected cell lysates, measured before the first vaccination (**B**) and 1 month after the second vaccination (**C**).

**Fig. S2.** **Immune marker expression profiles of DENV-2−specific CD4^+^ T-cell responses in Puerto Rican adults and adolescents.**

DENV-specific CD4^+^ T-cell responses in Puerto Rican adolescents and adults after immunization with the F17 or F19 vaccines are presented. Blood samples were obtained prior to each dose (Day [D] 0 and month [M] 6), 3 months after the first dose (M3) and 1 month after the second dose (M7). Data are presented in box-and-whiskers plots as percentages of DENV-specific CD4^+^ T cells expressing (after *in vitro* stimulation) at least the indicated immune marker (among IFN‑γ, IL-2, TNF‑α and CD40L) plus another, amongst all CD4^+^ T cells, with medians, first and third quartiles, and minimum/maximum values presented.

**Fig S3.** **DENV-specific memory B cells analyzed by B-cell ELISPOT.**

Participants were tested for the presence of DENV-specific memory B cells using B-cell ELISPOT. Peripheral blood mononuclear cells that were differentiated into antibody secreting cells were incubated in plates coated with either recombinant truncated 80E protein subunit of the respective DENV-serotype (to detect antigen-specific memory B cells), or with antihuman IgG (to detect total memory B cells). Background responses were assessed using uncoated plates. Antibody/antigen spots enumerating memory B cells were detected using a conventional immuno-enzymatic procedure, and data were expressed as frequencies of antigen-specific memory B cells within the total memory B-cell population. Representative wells are presented for one subject each of the placebo group (**A**) and the F19 vaccine group (**B**) of the US study, for blood samples collected at D180. Background responses (top rows), and responses after stimulation with DENV-1, DENV-2, DENV-3 and DENV-4 (bottom rows) are shown.
